# The dynamics of chromatin states mediated by epigenetic modifications during somatic cell reprogramming

**DOI:** 10.3389/fcell.2023.1097780

**Published:** 2023-01-16

**Authors:** Jing Peng, Wen Jie Zhang, Qi Zhang, Ying Hua Su, Li Ping Tang

**Affiliations:** State Key Laboratory of Crop Biology, College of Life Science, Shandong Agricultural University, Tai’an, Shandong, China

**Keywords:** somatic cell reprogramming, epigenetic modification, chromatin state, gene expression, cell pluripotency, cell totipotency

## Abstract

Somatic cell reprogramming (SCR) is the conversion of differentiated somatic cells into totipotent or pluripotent cells through a variety of methods. Somatic cell reprogramming also provides a platform to investigate the role of chromatin-based factors in establishing and maintaining totipotency or pluripotency, since high expression of totipotency- or pluripotency-related genes usually require an active chromatin state. Several studies in plants or mammals have recently shed light on the molecular mechanisms by which epigenetic modifications regulate the expression of totipotency or pluripotency genes by altering their chromatin states. In this review, we present a comprehensive overview of the dynamic changes in epigenetic modifications and chromatin states during reprogramming from somatic cells to totipotent or pluripotent cells. In addition, we illustrate the potential role of DNA methylation, histone modifications, histone variants, and chromatin remodeling during somatic cell reprogramming, which will pave the way to developing reliable strategies for efficient cellular reprogramming.

## Introduction

In mammals and plants, stem cells are undifferentiated cells with the capacity for self-renewal that also have potential to differentiate into multiple types of somatic cells with specific functions. Conversely, differentiated somatic cells can also be reprogrammed into totipotent or pluripotent states spontaneously or under specific inducing conditions, in a process called somatic cell reprogramming (SCR) (Ebrahimi et al., 2019). During SCR, genes expressed in somatic cells are shut down, while genes associated with totipotency or pluripotency are selectively activated (Surani, 2001). In mammals, SCR relies mainly on three general approaches: somatic cell nuclear transfer (SCNT), fusion with embryonic stem cells (ESCs), and transcription-factor-driven direct reprogramming. SCNT entails the transfer of a somatic nucleus into an oocyte whose nucleus has been removed, and involves five cellular events: nuclear membrane breakdown and formation of premature chromosome condensation (PCC); activation; nuclear expansion; DNA replication; and zygotic genome activation (ZGA) (Matoba and Zhang, 2018). The fusion of an ES cell (which is pluripotent) to a somatic cell can induce the somatic cell to revert back to a pluripotent state (Do and Schöler, 2004). In direct reprogramming, reprogramming factors that can reprogram the somatic cell into a pluripotent state are injected into somatic cells in a process consisting of three stages: initial effects on the somatic epigenome; transcriptional changes during early reprogramming; and induction and consolidation of pluripotency (Smith et al., 2016). In plants, SCR encompasses many approaches, such as somatic embryogenesis and regeneration. Somatic embryogenesis is a typical mode of SCR, whereby the somatic cell can be converted to a totipotent cell that can then give rise to an entire plant (Su et al., 2021). Plant regeneration is another representative example of SCR in plants, during which exposure of vegetative tissue to a combination of phytohormones induces the formation of relatively undifferentiated callus, which then regenerates new organs (Lee and Seo, 2018). The fundamental principle of SCR is the conversion of somatic cells into stem cells through rearrangement of epigenetic modifications affecting chromatin state (Lee and Seo, 2018), such as histone and DNA modifications, which strongly influence cell fate (Yao et al., 2020). There is mounting evidence that the chromatin state changes significantly to break down the barriers between diverse cell types through rebuilding of chromatin structures during SCR (Flottmann et al., 2012; Wang F. X. et al., 2020).

A large body of work has revealed that SCR can be triggered by the overexpression of certain essential transcription factor genes, which are recognized as totipotency or pluripotency genes. For instance, induced pluripotent stem cells (iPSCs) can form in mouse and human adult fibroblasts through the co-overexpression of a set of four genes referred to collectively as OSKM: *octamer-binding protein 4* (*OCT4*), *sex-determining region Y-box 2* (*SOX2*), *Krüppel-like factor 4* (*KLF4*), and *C-MYC* (Takahashi and Yamanaka, 2006; Takahashi et al., 2007). In plants, number of genes, when overexpressed, have been shown to have the capacity to reprogram somatic cells into totipotent state and form somatic embryos (SEs), including *LEAFY COTYLEDON1* (*LEC1*) (Lotan et al., 1998), *LEC2* (Stone et al., 2001), *BABY BOOM* (*BBM*) (Boutilier et al., 2002), *AGAMOUS-LIKE 15* (*AGL15*) (Harding et al., 2003), *RWP-RK DOMAIN-CONTAINING 4* (*RKD4*) (Waki et al., 2011), and *WUSCHEL* (*WUS*) (Su et al., 2009). Moreover, overexpression of some transcription factor genes can induce the formation of callus, a pluripotent cell mass, from somatic cells; these genes include *PLETHORA* (*PLT*) genes (Kareem et al., 2015), *WOUND INDUCED DEDIFFERENTIATION* (*WIND*) genes (Iwase et al., 2011), *WUSCHEL-RELATED HOMEOBOX 11* and *12* (*WOX11/12*) (Liu et al., 2014), and *LATERAL ORGAN BOUNDARIES DOMAIN 16* (*LBD16*) (Liu et al., 2018).

Although the changes in chromatin state mediated by both 1) epigenetic modifications and 2) the high-level expression of totipotency or pluripotency genes are important for SCR, our present knowledge of their complex and interwoven relationship is rather scarce. Recently, the deployment of transposase-accessible chromatin with high-throughput sequencing (ATAC-seq) has unraveled how the chromatin of these totipotency or pluripotency genes gradually opens during SCR, while that of somatic-cell-specific genes becomes gradually closed (Li et al., 2017; Wang F. X. et al., 2020). Intriguingly, the factors encoded by pluripotency genes can also directly interact with the components of chromatin-remodeling or chromatin-modifying complexes to regulate the dynamics of their own chromatin state, in a process that precedes transcriptional activation (Koche et al., 2011; Orkin and Hochedlinger, 2011). Here, we review the contribution of various epigenetic modifications, such as DNA methylation, histone modifications, histone variants, and chromatin remodeling, to the changes in chromatin state that occur during SCR in both plants and mammals, and present a comprehensive overview of the modifiers that facilitate or inhibit such reprogramming (Table 1).

**TABLE 1:** Epigenetic modifications and modifiers in somatic cell reprogramming.

Epigenetic modifications/modifiers		Biochemical function	Role for reprogramming	References
Plant	Mammal			
MET1	DNMT1	DNA methyltransferase	barrier for somatic cell reprogramming	Li et al. (2011); He et al. (2017); Shim et al. (2021b)
DRM1, DRM2, CMT3	DNMT3a/b	DNA methyltransferase	barrier for somatic cell reprogramming	Epsztejn-Litman et al. (2008); Guo et al. (2013); Shemer et al. (2015)
	TET1/2/3 TDG	DNA demethylase	essential for somatic cell reprogramming	Costa et al. (2013); Gao et al. (2013); Hu et al. (2014)
	RAD50	act as a Tet1-binding protein	facilitate pluripotent reprogramming	Park et al. (2020)
CLF, SWN, FIE		PRC2 components, catalyze H3K27me3 formation	the effect of reprogramming depending on the specific cell type	Makarevich et al. (2006); Bouyer et al. (2011); He et al. (2012); Ikeuchi et al. (2015)
	EZH2	PRC2 components, catalyze H3K27me3 formation	required for somatic cell reprogramming	Buganim et al. (2012); Onder et al. (2012); Ding et al. (2014)
	UTX	H3K27 demethylase	interact with OSK and facilitate somatic cell reprogramming	Mansour et al. (2012); Jiang et al. (2020)
	SUV39H1/H2, EHMT1/2 SETDB1	H3K9 methyltransferase	associated with transcriptional repression and inhibit somatic cell reprogramming	Lachner et al. (2001); Chen et al. (2013)
	KDM3/4	H3K9 demethylase	remove H3K9me3 to accelerate somatic cell reprogramming	Wei et al. (2017); Zhu et al. (2021)
	TRIM28	epigenetic modifier	interact with SETDB1 and repress the pluripotent genes expression to inhibit reprogramming	Miles et al. (2017)
	CBX3	A H3K9me3 reader	repress the pluripotency gene to block reprogramming	Sridharan et al. (2013)
KYP		H3K9 methyltransferase	promote shoot regeneration	Li et al. (2011)
JMJ30		H3K9 demethylase	interact with ARF7and ARF9 remove H3K9m3 at promoters of *LBD16* and *LBD29*	Lee et al. (2018)
ATX4		responsible for H3K4me3 accumulation	promote shoot formation	Lee et al. (2019)
ATXR2		confer H3K36me3 accumulation	ARF7-ARF9-JMJ30 complex recruit ATXR2 to accumulate H3K36me3, activate *LBD16* and *LBD29* to promote somatic cell reprogramming	Lee et al. (2017); Lee et al. (2021)
	WDR5	belong to MLL complex, a H3K4me3 reader	activate pluripotent genes expression and promote somatic cell reprogramming	Ang et al. (2011)
	ASH2L-b	a core component of MLL complex	improve reprogramming efficiency	Li et al. (2018)
	LSD1	catalyze the demethylation of H3K4me1/2	promote reprogramming by preventing H3K4 demethylation	Sun et al. (2016)
SDG8		H3K36me3 methyltransferase	accumulate H3K36me3 on *ASA1* and promote rooting from leaf explants	Zhang et al. (2019)
	JHDM1a/1b	H3K36 demethylase	interact with Oct4 to activate the microRNA 302/367, promote somatic cell reprogramming	Subramanyam et al. (2011); Wang et al. (2011)
	DOT1L	mediate H3K79 methylation formation	barrier for somatic cell reprogramming	Jones et al. (2008); Onder et al. (2012)
PRMT5	PRMT5	mediate H4R3sme2s formation	improve the efficiency of somatic cell reprogramming	Nagamatsu et al. (2011); Han et al. (2013); Liu et al. (2016)
GCN5, PRZ1	GCN5, TRRAP	responsible for histone methylation formation	form SAGA complex to activate reprogramming factors *via* catalyzing histone acetylation at gene loci, required for somatic cell reprogramming	Liu et al. (2003); Sieberer et al. (2003); Anzola et al. (2010); Hirsch et al. (2015); Kim et al. (2018); Rymen et al. (2019)
HAD6, HAD19		histone deacetylase	repress pluripotent genes expression	Tanaka et al. (2008); Zhou et al. (2013); Chhun et al. (2016)
	HDAC2, HDAC6	histone deacetylase	remove histone acetylation and cause pluripotent genes down-regulated	Wei et al., 2015; Sun et al., 2021
H3.15		replace canonical H3 and reduce the deposition of H3K27me3	promote callus formation	Yan et al. (2020)
H2A.Z		histone variant	act as a repressor of somatic cell reprogramming	Lambolez et al. (2022)
	H3.3	histone variant	act as a repressor of somatic cell reprogramming	Wen et al. (2014); Fang et al. (2018); Wang F. X. et al. (2020)
	macroH2A	overlap with H3K27me3, prevents the regain of H3K4me2	repress pluripotent genes expression	Pasque et al. (2012); Barrero et al. (2013); Gaspar-Maia et al. (2013); Pliatska et al. (2018)
SYD		a SWI2/SNF2-like protein in the SNF2 subclass	repress *WUS* expression	Kwon et al. (2005)
PKL, PKR2		CHD3 family of chromatin remodeling proteins	repress reprogramming factors by accumulation of H3K27me3 with PRC2	Dean Rider et al. (2003); Aichinger et al. (2009)
	NuRD	histone deacetylase activity and ATP-dependent nucleosome remodeling activity	act as a roadblock for SCR	Zhang et al. (1999); Luo et al. (2013); Rais et al. (2013)
	BRG1, BRF155	esBAF complex	increase H3K4me3and H3K9ac on promoter of reprogramming factors	Ho et al. (2009); Singhal et al. (2010); Ho et al. (2011); Jiang et al. (2015)
	HMGA1	HMG protein family	increase the rate of reprogramming	Kishi et al. (2012); Shah et al. (2012)
AHL15		result in heterochromatin decondensation	promote somatic embryogenesis	Karami et al. (2021)

## DNA methylation

DNA methylation is a classical epigenetic modification consisting of the addition of a methyl group to the cytosine base of DNA to form 5-methylcytosine (5 mC), which can occur at cytosine bases within all sequence contexts: symmetric CG and CHG and asymmetric CHH (where H = C, T, or A). DNA methylation can regulate gene expression by affecting chromatin structure, DNA conformation, DNA stability, and protein-DNA interaction (Dantas Machado et al., 2015). Recent evidence suggests that genome-wide changes in DNA methylation take place during SCR (Nishino et al., 2011; Li et al., 2019; Shim et al., 2021a). In mammals, the global methylation level of OSKM-iPSCs derived from different type cells (such as human endometrium or placental artery endothelium, among others) is significantly higher than that of their corresponding parental cell lines (Nishino et al., 2011; He et al., 2014). Intriguingly, some high-expression endogenous pluripotency genes [including *OCT4*, *Spalt Like Transcription Factor 4* (*SALL4*), *SOX8, zinc finger protein of the cerebellum 5* (*ZIC5*), and *Forkhead Box D1* (*FOXD1*)] show hypomethylation in iPSCs, because they contain regions specifically hypomethylated in stem cells that are available to be demethylated during reprogramming (Nishino et al., 2011). In addition, CG methylation is maintained by DNA methyltransferases (DNMTs). Knockdown of *DNMT1* by small heterochromatic RNA (shRNA) facilitates the generation of iPSCs at the early stage of reprogramming by decreasing the DNA methylation level of promoters from pluripotency genes [like *OCT4*, *NANOG*, *Estrogen Related Receptor Beta* (*ESRRB*), and *Developmental Pluripotency Associated 2* (*DPPA2*)] and thereby significantly increasing their expression (He et al., 2017). DNMT3a/b are responsible for *de novo* DNA methylation (Kinoshita et al., 2021). As with *DNMT1*, knockdown of *DNMT3a* or *DNMT3b* can improve the efficiency of iPSC generation, while the ectopic expression of *DNMT3a* or *DNMT3b* significantly inhibits reprogramming at the early stage, indicating that *DNMT3a* and *DNMT3b* expression acts as a barrier to cell reprogramming. Overexpression of the pluripotency factor gene *SOX2* can realize SCR through upregulating the expression of the microRNA gene *miR29b*, which can directly target *DNMT3a* and *DNMT3b* transcripts and repress their abundance (Guo et al., 2013). Furthermore, DNMT3a/b can be recruited by the histone methyltransferase G9a [also called Euchromatic histone-lysine N-methyltransferase 2 (EHMT2)] at pluripotency loci (*OCT3/4*, *NANOG*) to block the expression of these pluripotency genes through *de novo* DNA methylation, thus preventing reprogramming of differentiated mouse embryonic stem cells to a pluripotent state (Epsztejn-Litman et al., 2008).

Similarly, DNA methylation also dynamically changes during SCR in plants (Li et al., 2019; Shim et al., 2021a). The level of CHH methylation was reported to decrease during the leaf-to-callus transition in Arabidopsis (*Arabidopsis thaliana*) (Shim et al., 2021a). Several essential genes related to cell division have lower CHH methylation in callus than in leaf tissues, and their expression gradually increases upon auxin-triggered callus proliferation in Arabidopsis; these genes include *ORIGIN RECOGNITION COMPLEX 1* (*ORC1*), *REPLICATION FACTOR C 2* (*RFC2*), *MITOTIC ARREST DEFICIENT 1* (*MAD1*), and *DISRUPTION OF MEIOTIC CONTROL 1* (*DMC1*) (Shim et al., 2021a). Similarly, several genes involved in callus formation from leaf explants in strawberry (*Fragaria vesca*), such as *FvePLT3/7*, *FveWIND3*, *FveWIND4*, *LONELY GUY 4* (*FveLOG4*), and *INDOLE-3-ACETIC ACID INDUCIBLE 14* (*FveIAA14*), are upregulated during callus formation, and their expression is associated with reduced DNA methylation (Liu et al., 2022). CHH methylation levels also decrease during somatic embryogenesis in cotton (*Gossypium hirsutum*) (Li et al., 2019). Some phytohormone-related and *WUSCHEL*-related homeobox genes, such as *PIN-FORMED 1* (*PIN1*), *SMALL AUXIN UPREGULATED RNA 1* (*SAUR1*), *IAA14*, *IAA16*, *WUS4.1*, *PLT5/6*, and *ENHANCER OF SHOOT REGENERATION 12* (*ESR12*), are upregulated in conjunction with a decrease of CHH methylation level at their chromatin during somatic embryogenesis of cotton (Li et al., 2019). In plants, the DNA methyltransferase MET1 maintains CG methylation (Kankel et al., 2003), while CHROMOMETHYLASE 3 (CMT3) and CMT2 are involved in the maintenance of CHG methylation, and CMT2 and DOMAINS REARRANGED METHYLASE 2 (DRM2) are responsible for maintaining CHH methylation (Lindroth et al., 2001; Stroud et al., 2014). In addition, DRM1 and DRM2 catalyze *de novo* methylation *via* the RNA-directed DNA methylation (RdDM) pathway (Matzke and Mosher, 2014; Wendte and Pikaard, 2017). Loss of MET1 function or the *drm1 drm2 cmt3* triple mutant can induce shoot regeneration earlier than wild type in Arabidopsis (Li et al., 2011; Shemer et al., 2015) because of their lower DNA methylation level on the *WUS* promoter, thus shifting initial *WUS* expression earlier to reach a higher expression level compared with the wild type in the initial stage of shoot formation. In addition, the expression of the photoreceptor genes *CRYPTOCHROME 1* (*CRY1*) and *CRY2*, which can stimulate the expression of type-B ARR genes [including Arabidopsis *RESPONSE REGULATOR 1* (*ARR1*) and *ARR10*] and promote shoot regeneration, is higher in the *met1* mutant, with reduced CG methylation at their loci (Shim et al., 2021b). Application of 5-azacytidine (5-AzaC), an inhibitor of DNA methyltransferase, leads to an increased frequency of somatic embryo induction in some plant species [rapeseed (*Brassica napus*), barley (*Hordeum vulgare*), and Robusta coffee (*Coffea canephora*)], due to DNA hypomethylation (Solis et al., 2015). Likewise, 5-AzaC treatment facilitates the transformation of somatic cells into pluripotent cells during the late stages of direct reprogramming through the ectopic expression of defined transcription factor genes (*OCT4*, *SOX2*, *KLF4*, and *C-MYC*) in mouse (Mikkelsen et al., 2008). Importantly, all the above studies show that DNA methylation acts as a barrier during SCR in both mammals and plants, as most pluripotency- or totipotency-associated genes need to be hypomethylated before they can be activated.

Conversely, DNA methylation can also be cleared by DNA demethylases in a process called active DNA demethylation, which is thought to have a positive effect on SCR (Gao et al., 2013). In mammals, active DNA demethylation can be achieved *via* the independent action of TET (ten-eleven translocation) dioxygenases and TGDs (thymine DNA glycosylases). 5mC can be oxidized to 5-hydroxymethylcytosine (5hmC), 5-formylcytosine (5fC), and 5-carboxylcytosine (5caC) by TETs, after which 5fC and 5caC can be recognized in the genome and excised by TDGs, with the single-nucleotide gaps filled with unmethylated cytosine through the base-excision repair (BER) pathway (Wu and Zhang, 2017). TET1 has been shown to promote DNA demethylation at *OCT4* loci and reactivate its transcription at the early phase of iPSC induction (Gao et al., 2013). TET1 was also reported to be able to replace OCT4 in OSKM to initiate SCR and interact with NANOG to enhance the expression of pivotal pluripotency genes (such as *ESSRB* and *OCT4*) by removing 5mC from their promoters (Costa et al., 2013). Moreover, a triple knockout of *TET* genes (*TET1*, *TET2*, and *TET3*) or knockout of *TDG* genes can block the mesenchymal-to-epithelial transition (MET) to prevent SCR, suggesting that TET- and TDG-mediated active DNA demethylation is indispensable for the reprogramming of somatic cells (Hu et al., 2014). TET1 can also interact with Rad50 (a key player in DNA double-strand break repair) to facilitate active DNA demethylation at pluripotency genes (Park et al., 2020). In plants, the bifunctional 5-mC DNA glycosylase family REPRESSOR OF SILENCING 1 (ROS1), DEMETER (DME), DME-like 2 (DML2), and DML3 can remove 5mC from the genome (Bartels et al., 2018). The resulting single-nucleotide gaps are then filled with unmethylated cytosine through the BER pathway. Notably, very few demethylases have been reported to date in plants that affect cell reprogramming. In general, pluripotency genes are hypermethylated and transcriptionally silenced in somatic cells, and they must be reactivated through active DNA demethylation during SCR, whereas somatic genes must be silenced by *de novo* methylation to facilitate cell reprogramming (Ohi et al., 2011; Liang and Zhang, 2013; Haridhasapavalan et al., 2020; Poetsch et al., 2022). Therefore, during SCR, there may be a complex balance between DNA methylation and demethylation, which may occur simultaneously to alter the expression of somatic-cell-specific genes or pluripotency genes to promote cell reprogramming.

## Histone methylation

Histone methylation is one of the main mechanisms of epigenetic regulation. It is a dynamic and reversible reaction catalyzed by histone methyltransferases (HMTs) and histone demethylases (Bannister and Kouzarides, 2002). Methylation mainly targets lysine (K) and arginine (R) residues. Usually, chromatin can switch between open (euchromatin) and closed (heterochromatin) states (Allis, 2001). Heterochromatin comprises tightly packed, transcriptionally inactive regions of the genome and usually contains abundant methylation at specific histone sites [H3K9me3 (trimethylation of K9 on histone H3), H3K27me3, and H3K79me3]. By contrast, low folding and compression of euchromatin is necessary for transcriptional activation and is often associated with active histone methylation (like H3K4me3 and H3K36me3). This dynamic modification of histones drives the structural changes in chromatin conformation required for gene expression (Fuchs et al., 2006; Lawrence et al., 2016).

Polycomb repressive complex 2 (PRC2) is a key Polycomb Group (PcG)-type regulator complex catalyzing the deposition of H3K27me3, a repressive histone modification. PRC2, which has been identified as a key factor shaping epigenetic modifications in SCR, contains the proteins EZH1/2 (Enhancer of zeste homolog 1/2), EED (Embryonic ectoderm development), Su(z) 12 (Suppressor of zeste 12), and RBBP4/7 (Retinoblastoma binding protein 4/7) in humans (Bieluszewski et al., 2021). Their homologs in Arabidopsis are CURLY LEAF (CLF), SWINGER (SWN), MEDEA (MEA), FERTILIZATION INDEPENDENT ENDOSPERM (FIE), EMBRYO FLOWER 2 (EMF2), VERNALIZATION 2 (VRN2), FERTILIZATION INDEPENDENT SEED 2 (FIS2), and MULTICOPY SUPRESSOR OF IRA1 (MSI1) (Bieluszewski et al., 2021). In Arabidopsis, mutations in PRC2 components (such as FIE, CLF, and SWN) result in the formation of callus-like tissues or somatic embryos in roots due to the loss of H3K27me3 (Bouyer et al., 2011; Ikeuchi et al., 2015). PRC2 subunits can be directly targeted to the chromatin of the embryonic regulators *LEC2* and *FUS3* and the callus regulator *WIND3* in the vegetative-to-embryonic transition. Functional defects of PRC2 subunits lead to the removal of H3K27me3 at the promoter region of these target genes, thereby increasing their expression level (Makarevich et al., 2006; Bouyer et al., 2011; Ikeuchi et al., 2015). However, an opposite phenomenon was reported in *clf swn* leaf explants, which cannot form callus on callus-inducing medium (CIM) (He et al., 2012). Indeed, although leaf identity genes are repressed by PRC2-mediated H3K27me3 deposition in wild-type callus, they are highly expressed and prevent callus formation in the *clf swn* double mutant due to the lack of H3K27me3 (He et al., 2012), suggesting that H3K27me3 mediated by PRC2 is required for the leaf-to-callus transition. There is a different situation in mammals, where inhibition of *EZH2*, a core subunit of PRC2, led to a reduction in global H3K27me3 levels and iPSC production during early reprogramming (Buganim et al., 2012; Onder et al., 2012). EZH2 represses the *Ink4a/Arf* (inhibitor of CDK4/alternative reading frame) locus, which encodes a cell cycle inhibitor and acts as a ‘roadblock’ for the generation of iPSCs (Ding et al., 2014), suggesting that H3K27me3 deposition mediated by EZH2 is required for reprogramming of somatic cells toward pluripotency. H3K27me3 can be removed out by the H3K27 demethylase UTX [X-linked homologue of Uty, also named KDM6A (lysine demethylase 6A)]. Overexpression of *UTX* can facilitate iPSC reprogramming since UTX interacts with the pluripotency factor OSK and binds to the promoters of pluripotency genes (*SALL1*, *SALL4*, and *UTF1*) to promote their expression through its histone demethylase catalytic activity during early reprogramming (Mansour et al., 2012; Jiang et al., 2020). However, elimination of H3K27me3 does not always have a positive effect on SCR, as overexpression of another H3K27 demethylase gene, *KDM6B*, impaired genetic reprogramming during SCNT (Mansour et al., 2012; Jiang et al., 2020). Therefore, only erasure of H3K27me3 at certain loci is required for reprogramming, which also indicates that SCR is regulated by H3K27me3 *via* an elaborate mechanism.

The repressive mark H3K9me3 acts as an important barrier to SCR and is abundant at repressive chromatin regions. The H3K9 methyltransferase SUV39H1 is required for the maintenance of these H3K9me3 domains through recruitment of heterochromatin proteins (HP1α, HP1β, and HP1γ) to establish heterochromatin and silence gene expression (Lachner et al., 2001). Inhibition of H3K9 methyltransferases [SUV39H1, SUV39H2, SETDB1 (SET domain, bifurcate 1), EHNT1/GLP, and EHMT2/G9a, all belonging to the KMT1 (lysine methyltransferase 1) class] expression promotes the generation of iPSCs (Onder et al., 2012; Chen et al., 2013), as knockdown of *SETDB1* by short interfering RNA (siRNA) is sufficient to convert pre-iPSCs into iPSCs by reducing H3K9 methylation levels at core pluripotent loci (such as *NANOG*, *OCT4*, and *SOX2*) (Chen et al., 2013). Moreover, SETDB1 can also interact with the epigenetic modifier TRIM28 (Tripartite motif-containing 28), by which it is recruited to establish the repressive epigenetic marks H3K9me2/3 and keep endogenous retroviruses (ERVs) silenced (Miles et al., 2017). The reduction of TRIM28 expression can enhance reprogramming by increasing the expression of genes located in repressive chromatin regions, such as *NANOG*, *LIN28b* (*Lin-28 Homolog b*), *ESRRB*, *FGF4* (*Fibroblast Growth Factor 4*), *OCT4*, and *SOX2* (Miles et al., 2017), suggesting that TRIM28 also acts as a barrier for cell reprogramming. Similarly, inhibition the expression of heterochromatin-protein-1 γ (Cbx3), which is an H3K9me3 reader associated with active transcription, facilitates reprogramming. Indeed, H3K9 methyltransferases and Cbx3 were reported to repress the pluripotency gene *NANOG* to block reprogramming (Sridharan et al., 2013). In plants, a similar mechanism has been described for H3K9me3 as a barrier to SCR. Mutation in the *KRYPTONITE* (*KYP*) gene encoding a histone H3K9 methyltransferase was reported to promote *in vitro* shoot organogenesis by increasing *WUS* expression (Li et al., 2011). Conversely, overexpression of *KDM4b*, an H3K9 demethylase gene, can significantly improve the efficiency of embryonic stem cell reprogramming in cloned mouse embryos by a specific reduction in H3K9/36me3 levels and upregulation of core pluripotency genes in mouse embryonic fibroblasts (Wei et al., 2017). KDM3a and KDM3b also cooperate with OCT4–SOX2 to maintain the pluripotency gene regulatory network *via* their demethylase activity (Zhu et al., 2021). In plants, a knockout in the histone demethylase gene *JUMONJI C DOMAIN-CONTAINING PROTEIN 30* (*JMJ30*) inhibited the formation of callus from leaf explants in Arabidopsis (Lee et al., 2018). JMJ30 can bind to the promoter of *LBD16* and *LBD29* along with AUXIN RESPONSE FACTOR 7 (ARF7) and ARF19, and remove the repressive H3K9me3 mark from the promoter of these genes during leaf-to-callus transition (Lee et al., 2018). The balance between H3K9 methylation and demethylation may provide a dynamic switch between heterochromatin and euchromatin to specify cell fate during reprogramming.

In contrast, the H3K4 and H3K36 marks usually act as activators during SCR. In Arabidopsis, ARABIDOPSIS TRITHORAX 4 (ATX4), which is responsible for the accumulation of H3K4me3 at target loci, has been reported to promote *in vitro* shoot organogenesis (Lee et al., 2019). The *atx4-2* mutant showed enhanced callus formation during leaf-to-callus transition but a reduced shoot formation capability during callus-to-shoot regeneration due to the downregulation of several shoot identity genes [*HOMEOBOX GENE 1* (*ATH1*), *SAWTOOTH 1* (*SAW1*), *SAW2*, *TCP DOMAIN PROTEIN 10* (*TCP10*), and *YABBY5* (*YAB5*)], which is accompanied by reduced H3K4me3 accumulation at these genes (Lee et al., 2019). In addition, ARABIDOPSIS TRITHORAX-RELATED 2 (ATXR2), a histone lysine methyltransferase, can promote H3K36me3 accumulation and facilitate callus formation from leaf explants through activating *LBD* genes (Lee et al., 2017). Moreover, ATXR2 interacts with ARR1 to facilitate H3K36me3 deposition at type-A *ARR5* and *ARR7* loci and activate their expression, resulting in lower *WUS* expression and a failure of *de novo* shoot regeneration (Lee et al., 2021). These results indicate that a single histone modifier, ATXR2, can exhibit distinct functions in callus formation and shoot regeneration. In mammals, the Set/MLL HMT complex subunit, the H3K4me3 reader WDR5 (WD-repeat protein-5), is required for the formation of iPSCs through their H3K4 methylase catalytic activity (Ang et al., 2011). A decrease in *WDR5* expression causes a global reduction of H3K4me3 levels and resulting downregulation of pluripotency gene expression (such as *OCT4*, *SOX2*, *KLF4*, *NANOG*, and *ESRRB*) at the initiation phase (Ang et al., 2011). Moreover, WDR5 can interact with OCT4 to promote the expression of pluripotency genes (such as *OCT4*, *NANOG*, and *SOX2*) by establishing the H3K4me3 mark at the promoters of these genes (Ang et al., 2011). Furthermore, *WDR5* and another core component of the MLL complex, *ASH2L-b* (*absent, small, or homeotic discs 2-like b*), are downstream targets of OCT4, and their expression increases with higher OCT4 protein stability, resulting in higher H3K4me3 levels and greater efficiency of pluripotency induction in mouse embryonic fibroblasts (Li et al., 2018). Furthermore, suppression expression of the gene encoding Lysine-specific histone demethylase 1 (LSD1/KDM1a), which catalyzes the demethylation of H3K4me1/2, also promotes reprogramming by facilitating the expression of exogenous transcription factor genes like *OCT4*, *KLF4*, and *SOX2* at the early stage of SCR (Sun et al., 2016), suggesting that the H3K4me3 mark plays a positive role in SCR in most cases. Another histone lysine methyltransferase, SET DOMAIN GROUP8 (SDG8), augments H3K36me3 at the *ANTHRANILATE SYNTHASE α1* (*ASA1*) locus, a tryptophan biosynthesis gene that participates in the auxin biosynthesis pathway. SDG8-mediated H3K36me3 leads to increased *ASA1* expression, which can promote auxin biosynthesis and thus enhance root regeneration from leaf explants in Arabidopsis (Zhang et al., 2019). These results suggest that the H3K36me3 mark plays an important role in improving plant regeneration capability and efficiency. Nevertheless, unlike in plants, the H3K36 methylation mark acts as a barrier for SCR in mammals. The histone demethylases JHDM1a and JHDM1b, two known vitamin C-dependent H3K36 demethylases, can promote the generation of iPSCs by eliminating the H3K36me3 mark from the promoters of pluripotency genes, such as *CDH1* (*Cadherin-1*), *DSP* (*Desmoplakin*), and *IRF6* (*Interferon Regulatory Factor 6*), which are early responsive genes that contribute to the activation of the pluripotency gene *NANOG* during early reprogramming (Liang et al., 2012). Moreover, JHDM1b also interacts with OCT4 to activate the microRNA cluster miR302/miR367, which plays an essential role in maintaining the cell cycle in ESC and enhances reprogramming of mouse embryonic fibroblasts into iPSCs (Subramanyam et al., 2011; Wang et al., 2011). Therefore, in mammals, removal of H3K36me2/3 is beneficial for SCR. Likewise, H3K79 methylation has also been considered to act as a roadblock during SCR. H3K79 methylation mediated by DOT1L (Disruptor of telomeric silencing 1-like), a histone methyltransferase specific for H3K79, is associated with heterochromatin formation and embryonic development (Jones et al., 2008; Onder et al., 2012). Knockdown of *DOT1L via* shRNA increases the number of iPSCs along with a decrease in global H3K79 methylation levels (Onder et al., 2012). Furthermore, DOT1L inhibition can replace Klf4 or C-MYC to raise the expression of pluripotency genes (like *NANOG* and *LIN28*) in the early to middle stages of reprogramming and accomplish SCR (Onder et al., 2012). These results suggest that removal of H3K79 methylation is also beneficial for SCR.

Histone arginine methylation also has been shown to affect the efficiency of SCR. In Arabidopsis, the loss of function of PROTEIN ARGININE METHYLTRANSFERASE 5 (PRMT5), which is responsible for the formation of symmetric dimethylation of histone H4R3 (H4R3sme2s), reduces the efficiency of shoot regeneration (Liu et al., 2016). Indeed, PRMT5 can inhibit the expression of *KIP-RELATED PROTEIN*s (*KRP*s), which act as a repressor of cell cycle, and the levels of the H4R3sme2 modification at the *KRP1* and *KRP2* promoter regions are lower in Arabidopsis *prmt5* mutant, resulting in increased *KRP1* and *KRP2* transcript levels (Liu et al., 2016). In mammals, PRMT5 can cooperate with the pluripotency factors KLF4 and OCT3/4 early on to improve the efficiency of SCR (Nagamatsu et al., 2011). PRMT5 can also regulate L-threonine dehydrogenase (TDH) activity through its methyltransferase activity and interact with TDH to enhance SCR efficiency (Han et al., 2013). In brief, histone methylation is required for SCR. Different histone methylations are characteristic of open or repressive chromatin states, ensuring the activation of pluripotency genes to promote SCR.

## Histone acetylation

Histone acetylation and deacetylation are catalyzed by histone acetyltransferases (HAT) and histone deacetylases (HDAC), respectively, and are essential epigenetic marks that can regulate gene expression by changing the chromatin state and determine the direction of stem cell differentiation (Seto and Yoshida, 2014). Unlike histone methylation, lysine acetylation represents an open chromatin state closely related to transcriptional activation, whereas lysine deacetylation represents a repressive chromatin state that is typical of transcriptional repression (Shahbazian and Grunstein, 2007).

Previous studies have indicated that high histone acetylation at pluripotency genes can open their chromatin to facilitate cellular reprogramming (Zhang and Laux, 2018; Li et al., 2020). In Arabidopsis, HISTONE ACETYLTRANSFERASE OF THE GNAT/MYST SUPERFAMILY 1 (HAG1), also known as General control non-repressed protein 5 (GCN5), has been reported to play a vital role in the acquisition of pluripotency during shoot regeneration (Kim et al., 2018; Rymen et al., 2019). GCN5-mediated histone acetylation is highly enriched at the transcription start sites (TSS) of pluripotency genes, including *WOX5*, *WOX14*, *SCARECROW* (*SCR*), *PLT1*, *PLT2*, *WIND1*, *ETHYLENE-RESPONSE FACTOR 113* [*ERF113*, also called *RELATED TO APETALA2.6 L* (*RAP2.6 L*)], and *LBD16*, providing an open chromatin state for their transcriptional activation during early shoot induction (Kim et al., 2018; Rymen et al., 2019). PROPORZ1 (PRZ1), also known as ADA2b (transcriptional ADAptor 2b), is a transcriptional adaptor and a subunit of the Spt-Ada-Gcn5-acetyltransferase (SAGA) complex, which is associated with histone acetylation activity mediated by GCN5 (Grasser et al., 2021). PRZ1 was suggested to modulate GCN5 activity, thus promoting the accumulation of histone acetylation and the expression of target genes (Grasser et al., 2021). Mutation in *PRZ1* triggers the formation of tumorous callus-like tissue on *prz1-1* roots, due to its failure to convert auxin signals into proper morphogenic signals for lateral root formation (Sieberer et al., 2003). This defective growth response arises from changes in the expression of core cell cycle regulator genes such as *KRP*, which encodes an inhibitor of CYCLIN DEPENDENT KINASE (CDK) and is downregulated in the *prz1-1* mutant. These results suggest that histone acetylation acts as a positive regulator of gene expression and is required for SCR (Sieberer et al., 2003; Anzola et al., 2010).

Similarly, the GCN5-mediated SAGA complex is also a critical regulator of reprogramming initiation in mammals (Hirsch et al., 2015). Loss of GCN5 or at least two of the three other components of the SAGA complex [CCDC101 (coiled-coil domain containing 101), TAF12 (TATA-Box Binding Protein Associated Factor 12), and ATXN7L3 (Ataxin 7 Like 3)] leads to downregulation of RNA splicing and processing genes [such as *SNRPD1* (*Small Nuclear Ribonucleoprotein D1*), *SKIV2L2* (*Superkiller viralicidic activity 2-like 2*), *PRPF4* (*Pre-MRNA Processing Factor 4*), *PNN* (*Pinin*), *ISY1* (*Interactor of SYf1*), *U2AF1* (*U2 Small Nuclear RNA Auxiliary Factor 1*), and *SNRPG* (*Small Nuclear Ribonucleoprotein Polypeptide G*)] and decreased cell proliferation or survival, thus reducing SCR efficiency (Hirsch et al., 2015). Furthermore, the transcription factor MYC can directly activate *GCN5* and the other components of the SAGA complex in mouse ESCs to initiate a positive transcriptional feedback loop. In addition, MYC and GCN5 can also co-regulate a group of genes related to RNA splicing and RNA processing, which is essential for SCR (Zavolan and Kanitz, 2018). During SCR, GCN5 can be recruited by TRRAP (transformation-transactivation domain-associated protein) to MYC for transcriptional activation *via* its acetylase activity (Liu et al., 2003). Together, these data suggest that histone acetylation acts as an epigenetic activator to reprogramming by establishing a chromatin structure that promotes the activation of a transcriptional network and the regulation of pluripotency during early SCR.

Numerous studies have shown that inhibition of histone deacetylation significantly enhanced the reprogramming of somatic cells into pluripotent or totipotent cells (Zhang and Wu, 2013; Wojcikowska et al., 2018; Bie et al., 2020; Lee et al., 2020; Yang et al., 2021). Blocking HDAC activity with the histone deacetylase inhibitors trichostatin A (TSA) or sodium butyrate (NaB) promoted SCR in plants and mammals (Zhang and Wu, 2013; Wojcikowska et al., 2018; Bie et al., 2020; Lee et al., 2020; Yang et al., 2021). In plants, TSA treatment can induce the formation of somatic embryos from explants without the exogenous application of auxin by upregulating the expression of *YUCCA* auxin biosynthesis genes (*YUC1* and *YUC10*) and the pluripotency genes *LEC1*, *LEC2*, *FUSCA 3* (*FUS3*), *BBM*, and *AGL15*, possibly due to increased histone acetylation at these genes (Wojcikowska et al., 2018). NaB was also reported to enhance adventitious shoot formation of *Nicotiana benthamiana* in a concentration-dependent manner (Lee et al., 2020). A low concentration of NaB exerts a significant effect in stimulating adventitious shoot formation in calli derived from *N. benthamiana* protoplasts, which was accompanied by increased histone H3 acetylation (Lee et al., 2020). A double RNA interference (RNAi) line of the histone deacetylase genes *HDA6* and *HDA19* displayed embryo-like structures on true leaves along with high expression levels of *LEC1*, *FUS3*, and *ABSCISIC ACID-INSENSITIVE 3* (*ABI3*) in Arabidopsis, suggesting that HDA6 and HDA19 act to inhibit embryo-specific transcription factor gene expression and the embryogenic program (Tanaka et al., 2008). VP1 (Viviparous1)/ABI3-LIKE 1 (VAL1) and VAL2 may also serve as factors that recruit HDA6 and HDA19 to *LEC* promoters and repress their transcription (Zhou et al., 2013; Chhun et al., 2016). In mammals, as in plants, HDAC-mediated histone deacetylation acts as a barrier for SCR. Knockdown of *HDAC2* has been shown to efficiently improve OSKM-mediated iPSC generation, since loss of HDAC2 increases histone acetylation and enhances TET1 binding activity and DNA demethylation at the promoters of iPSC maturation genes during pre-iPS cell maturation (Wei et al., 2015). Inhibition the expression of HDAC6 can upregulate *OCT4* and *CDX2* (*Caudal Type Homeobox 2*) and raise the efficiency of SCNT embryo development by increasing histone H3K9/K14 and H4K8 acetylation levels (Sun et al., 2021). Overall, HDACs-mediated histone deacetylation can change the chromatin structure and make the chromatin inaccessible for transcription.

## Histone variants

Histone variants are atypical proteins that are highly similar to conventional histones. Histone variants have been shown to affect nucleosome stability and change the chromatin state by replacing canonical histones (containing histones H2A, H2B, H3, H4) (Talbert and Henikoff, 2017). In alfalfa (*Medicago sativa*), expression of the histone variant genes *H3-1* and *H3-11* was detected during somatic embryogenesis, but the underlying regulatory mechanisms remain unclear (Kapros et al., 1992). A recent study has shown that the histone variant H3.15 can promote callus formation in Arabidopsis (Yan et al., 2020). Indeed, H3.15 can replace canonical H3s and reduce the deposition of H3K27me3 catalyzed by PRC2, resulting in transcriptional derepression of downstream genes such as *WOX11* and *LBD18* (Yan et al., 2020). Another histone variant, H2A.Z, acts as a repressor of SCR in Arabidopsis, as a double mutant lacking two HISTONE H2A.Z variant genes, *hta9 hta11*, displayed enhanced shoot regeneration (Lambolez et al., 2022). Some genes involved in organ regeneration or auxin biosynthesis, such as *YUC* genes, are upregulated by the reduction of H2A.Z that occurs when plants are exposed to high temperature (27 °C) (Lambolez et al., 2022). Furthermore, H2A.Z is highly conserved in eukaryotes and its abundance is enriched near the TSS of genes with high transcriptional activity (Dong et al., 2016; Zhang et al., 2017). These results suggest that H2A.Z may be involved in modulating chromatin structure to enhance access of transcription factors to genes critical for pluripotency and conducive to reprogramming (Dong et al., 2016; Zhang et al., 2017). Another histone variant, H3.3, which can replace canonical H3s, carries the repressive histone modification H3K27me3 and contributes to the accumulation of H3K4me3 and H3K36me3 (Wen et al., 2014; Fang et al., 2018). H3.3 deposition leads to the acquisition of cell pluripotency at the late stage of SCR in mammals (Wen et al., 2014; Fang et al., 2018). Moreover, knockdown of the H3.3 genes causes the downregulation of pluripotency genes (*OCT4* and *SOX2*) and an increase in H3K9me3, which in turn represses the reprogramming potential and efficiency of somatic cell nuclear transfer (SCNT) embryos (Wang Y. et al., 2020). However, the histone variant macroH2A acts as a barrier to the generation of iPSCs in mammals. Removal of macroH2A enhanced reprogramming efficiency (Pasque et al., 2012; Barrero et al., 2013; Gaspar-Maia et al., 2013; Pliatska et al., 2018). MacroH2A preferentially occupies genes marked with H3K27me3, such as *OCT4* and *NANOG*, and prevents the regeneration of H3K4me2 at the early stage of reprogramming (Barrero et al., 2013; Gaspar-Maia et al., 2013). Thus, most histone variants change the chromatin state by replacing canonical histones and then blocking histone modifications to prevent the reactivation of critical pluripotency genes during SCR.

## Chromatin remodeling

Chromatin remodeling complexes alter the nucleosome distribution at specific loci and the chromatin structure to facilitate the access of transcription factors to their cognate DNA sequences (Clapier and Cairns, 2009). ATP-dependent chromatin remodeling complexes consist of four major subfamilies: switch/sucrose non-fermentable (SWI/SNF), chromodomain helicase DNA-binding (CHD), imitation switch (ISWI), and inositol requiring 80 (INO80) (Luo et al., 2013; Cabot and Cabot, 2018; Mashtalir et al., 2018; Song et al., 2022). These chromatin remodeling complexes contain multiple protein subunits, utilizing ATP hydrolysis to restructure the nucleosome and ultimately change the chromatin state (Clapier and Cairns, 2009). In mammals, the esBAF (found in embryonic stem cells) complex possesses a unique subunit composition not found in other cell types, defined by the presence of BRG1 (Brahma-related gene 1), BAF155 (BRG1-Associated Factor 155), and BAF60a and the absence of BRM (Brahma), BAF170, and BAF60c, which are present in somatic cells (Ho et al., 2009). Continuous overexpression of *BRG1* and *BAF155* or knockdown of *BRM* and *BAF170* can facilitate the production of iPSCs induced by OSKM (Singhal et al., 2010; Jiang et al., 2015). The esBAF complex acts *via* the STAT3 (signal transducer and activator of transcription 3) signaling pathway, which can prevent cell differentiation and plays an essential role in pluripotency by binding to the chromatin of pluripotency genes (Ho et al., 2011). BRG1 can establish chromatin accessibility at STAT3-binding target genes to help STAT3 bind to these promoters by opposing PcG-mediated H3K27me3 deposition (Ho et al., 2011; Jiang et al., 2015). Moreover, BRG1 and BAF155 can also increase the H3K4me3 and H3K9ac marks, and reduce DNA methylation of the promoters of these pluripotency genes (OCT4, SOX2, NANOG). BRG1 and BAF155 can also interact with OCT4 to enhance its binding to downstream pluripotency genes such as *SALL4*, *DPPA4* (*Developmental Pluripotency Associated 4*), and *OCT4* (Ho et al., 2009; Ho et al., 2011). These results provide a possible explanation of the requirement for esBAF complexes in pluripotency.

In Arabidopsis, mutation in the SPLAYED (SYD) component of the SWI2/SNF2 complex exhibits defect in the maintenance of shoot apical stem cells, as SYD is recruited to establish a euchromatic state at the promoter of the pluripotency regulator gene *WUS* to transcriptionally regulate its expression (Kwon et al., 2005). Therefore, the BAF chromatin remodeling complex may play an essential role in the maintenance of the transcriptional program by regulating chromatin structure. By contrast, another type of chromatin remodeling factor, the CHD3 proteins PICKLE (PKL) and PICKLE-RELATED 2 (PKR2), are functionally redundant and prevent the formation of somatic embryos (Dean Rider et al., 2003; Aichinger et al., 2009). Mutation in *PKL* or *PKR2* results in tissues with embryonic traits and can cause reduced H3K27me3 levels and, thus, increased expression of the pluripotency genes *LEC1*, *LEC2*, and *FUS3* (Dean Rider et al., 2003; Aichinger et al., 2009). In addition, the *pkl* mutant can also enhance the phenotype of the *clf swn* double mutant, which produces SEs from seedlings (Aichinger et al., 2009). PKL has been shown to bind to *EMF2* and *SWN* promoters, and loss of PKL function reduces the expression and H3K27me3 levels of these *PRC2* genes (Aichinger et al., 2009). Thus, PKL may repress pluripotency genes by directly activating the accumulation of H3K27me3 at these PRC2 genes. These results reveal that chromatin remodeling mediated by CHD may impose a repressive chromatin state that prevents the transcription of pluripotency genes during SCR.

Similarly, overexpression of genes encoding subunits of the nucleosome remodeling and deacetylase (NuRD) complex can interfere with the reprogramming of somatic cells into iPSCs in mammals (Luo et al., 2013). The complex contains multiple subunits, including the ATPase Mi-2 (auto-antigen for dermatomyositis), HDAC1/2, Mta1/2 (Metastasis-associated protein1/2), and MBD2/3 (methyl-binding domain proteins 2/3) (Cabot and Cabot, 2018). The NuRD complex binds to methylated DNA, which it demethylates to repress transcription *via* the formation of heterochromatin (Zhang et al., 1999). Depletion of MBD3 triggers the transcriptional activation of pluripotency genes and enhances the production of iPSCs, even in the absence of C-MYC or SOX2 (Luo et al., 2013; Rais et al., 2013). Furthermore, overexpression of *MBD3* causes the establishment of heterochromatic features and the silencing of pluripotency genes (including *OCT4* and *NANOG*) in the late stage of reprogramming (Luo et al., 2013). Similarly, MBD3 can be directly recruited to the downstream target genes of OSKM, which are required for multiple reprogramming processes, to prevent their reactivation (Luo et al., 2013). Thus, the NuRD complex acts as a roadblock for SCR.

## Others

The high-mobility group (HMG) protein family is a type of non-histone chromatin binding protein that participate in transcriptional regulation, RNA processing, and chromatin states (Sgarra et al., 2010). The genes encoding HMG group A (HMGA) proteins were highly expressed and their encoding proteins were highly abundant during embryogenesis and can bind to AT-rich regions (also called AT-hook motifs) to regulate the chromatin state and gene expression (Pfannkuche et al., 2009). HMGA proteins can also regulate the global chromatin state and are required for open chromatin in neural precursor cells early in the reprogramming of mammalian cells (Kishi et al., 2012; Shah et al., 2012; Karami et al., 2021). Overexpression of *HMGA1* increased the reprogramming efficiency of somatic cells into iPSCs through HMGA1 binding to the promoters of pluripotency genes (*SOX2*, *LIN28*, and *C-MYC*) to induce their expression (Shah et al., 2012). Similarly, overexpression of the HMGA protein family gene *AT-HOOK MOTIF CONTAINING NUCLEAR LOCALIZED 15* (*AHL15*) resulted in heterochromatin decondensation and somatic embryogenesis in Arabidopsis (Karami et al., 2021). Notably, knockdown of *GhHmgB3*, a member of the high-mobility group box (HMGB) family genes, failed to generate somatic embryos in cotton (Hu et al., 2011). In conclusion, HMG proteins may increase the expression of pluripotency genes through chromatin opening, thus promoting the occurrence of SCR.

## Concluding remarks and future directions

**FIGURE 1:**
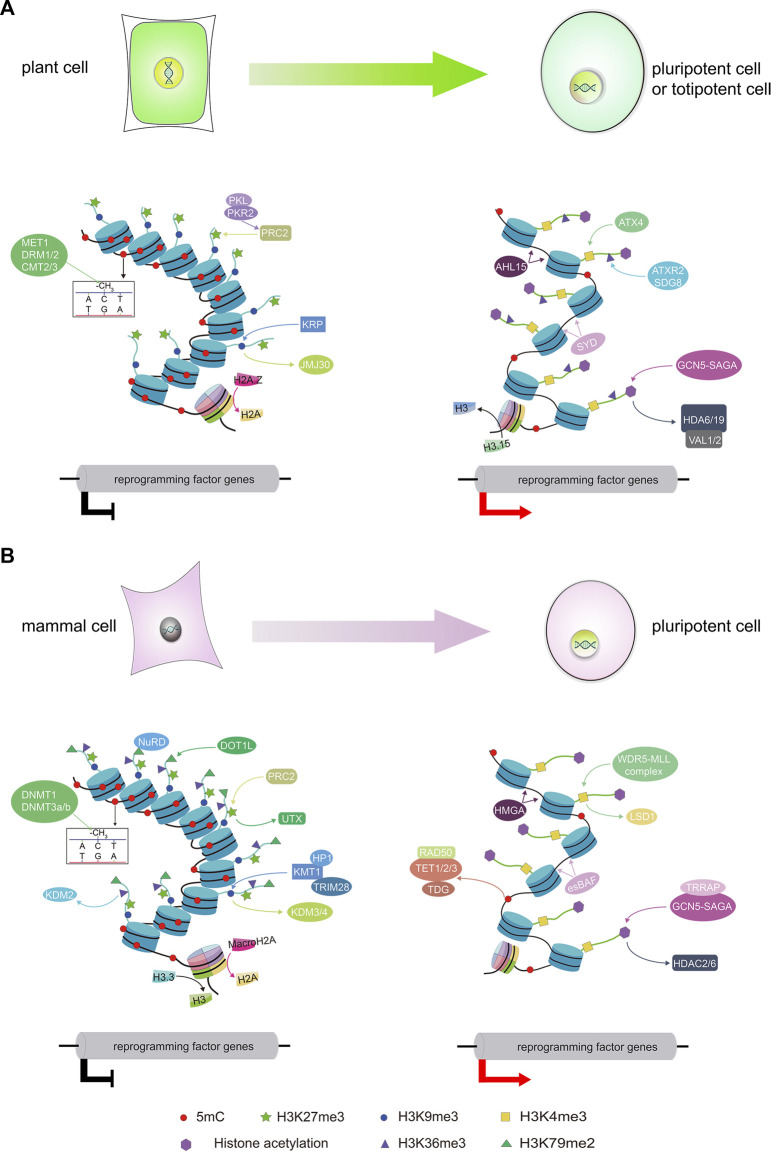
An overview of epigenetic factors dynamically regulating the chromatin state during somatic cell reprogramming. The diagrams illustrate the reprogramming of somatic cells to pluripotent or totipotent cells along with the associated epigenetic modifications in plants **(A)** and mammals **(B)**. DNA methylation acts as a barrier during SCR in both mammals and plants. Most pluripotency or totipotency genes are hypermethylated and transcriptionally silenced in somatic cells, and they are (re) activated through active DNA demethylation during SCR. Histone methylation is also required for SCR. Different histone methylation marks induce open or repressive chromatin states, ensuring the activation of pluripotency or totipotency genes to promote SCR. Histone acetylation acts as an epigenetic activator of reprogramming by establishing a chromatin structure that promotes the activation of a transcriptional network that regulates pluripotency or totipotency. Histone variants and chromatin remodeling are also associated with histone modifications and impose a repressive chromatin state to prevent the reactivation of critical pluripotency or totipotency genes during SCR.

SCR is a breakthrough for basic biology and has broad applications. In the medical field, SCR can turn somatic cells from patients into stem cells, which might then be used for tissue and cell therapies and even organ transplant. In plant biology, SCR can be used for rapid propagation, obtaining virus-free shoots, and assisting crop production. The ectopic expression of combinations of pluripotency factor genes has been pioneered to induce SCR, and different epigenetic factors can be integrated into the pluripotency factor network at multiple levels to accelerate reprogramming. Although several recent mechanistic studies have revealed that epigenetic modifications alter the chromatin state to regulate SCR (Figure 1), we still know very little about the role of epigenetics in SCR. As discussed above, the epigenetic modification H3K27me3 may act as a barrier for somatic embryogenesis but may be required for the leaf-to-callus transition in Arabidopsis. In addition, treatments with epigenetic-related small molecule inhibitors such as 5-AzaC or TSA can accelerate SCR, underscoring the need to clarify the function of these epigenetic factors in various cell types and different stages of SCR. Understanding the dynamic changes underlying epigenetic modifications and chromatin states can improve reprogramming efficiency and enable the generation of genetically stable pluripotent or totipotent cells. Furthermore, with the rapid development of single-cell transcriptome deep sequencing (scRNA-seq) technologies, single-cell ATAC-seq, and single-cell epigenomics, we are in a position to uncover which somatic cells can be reprogrammed into pluripotent or totipotent cells and what changes in gene expression, chromatin status, and epigenetic modifications accompany this cellular transition, which might shed light on the epigenetic regulatory mechanisms of cell pluripotency and totipotency. Moreover, locus-specific manipulation of epigenetic modifications through epigenetic editing and engineering could enhance the efficiency of SCR, which may be advantageous for applications in the fields of medicine and precision breeding in agriculture.
